# Supplementary material for: Mapping the evolution of fertility support policies in China: A content and instrumental analysis
Source: PLoS One. 2025 Oct 9;20(10):e0332137. doi: 10.1371/journal.pone.0332137 (PMC12510515; doi:10.1371/journal.pone.0332137)
Supplement: S1 Appendix — (ZIP) [file pone.0332137.s001.zip › S1 Appendix. 226 original policy documents/104-全国人民代表大会常务委员会办公厅关于“十四五”规划纲要编制工作若干重要问题专题调研工作情况的报告(FBM-CLI-1-5055217).doc]

全国人民代表大会常务委员会办公厅关于“十四五”规划纲要编制工作若干重要问题专题调研工作情况的报告

**发布部门：** [全国人大常委会办公厅](https://www.pkulaw.com/law/chl?Aggs.IssueDepartment=202)

**发布日期：**2020.08.10

**实施日期：**2020.08.10

**时效性：** [现行有效](https://www.pkulaw.com/law/chl?Aggs.TimelinessDic=01)

**效力级别：** [工作文件](https://www.pkulaw.com/law/chl?Aggs.EffectivenessDic=XA0105)

**法规类别：** [人大议事](https://www.pkulaw.com/law/chl?Aggs.Category=00106) [营商环境优化](https://dobusiness.pkulaw.com/)

全国人民代表大会常务委员会办公厅关于“十四五”规划纲要编制工作若干重要问题专题调研工作情况的报告
--2020年8月10日在第十三届全国人民代表大会常务委员会第二十一次会议上
全国人大常委会副秘书长 信春鹰

全国人民代表大会常务委员会：
　　根据《全国人大常委会2020年度工作要点》和《[全国人大常委会2020年度监督工作计划](https://www.pkulaw.com/chl/5c087e401d321375bdfb.html)》，全国人大常委会围绕“十四五”规划纲要编制工作若干重要问题开展专题调研。从3月初到7月中旬，常委会办公厅和有关专门委员会、工作委员会精心组织、周密安排、协调配合、加紧工作，圆满完成了调研任务，取得积极成果。受委员长会议委托，现将专题调研工作有关情况报告如下。
　　**一、**开展专题调研工作的基本情况
　　编制“十四五”规划纲要，是今年党和国家的一项重要任务。为了服务党和国家工作大局，贯彻落实中央重大决策部署，全国人大常委会高度重视，围绕“十四五”规划纲要编制若干重要问题开展专题调研，为明年十三届全国人大四次会议审查批准“十四五”规划纲要做好准备。栗战书委员长作出重要指示：认真做好“十四五”规划编制工作若干重要问题的专题调研。要抓紧制定调研方案，把题目选精、选实，契合今后五年经济社会发展的重点难点问题。考虑到当前疫情防控形势，组织调研活动要更加注重统筹、细化安排，深入基层实际，摸清情况和问题。提出意见建议要具体化、可操作，不能泛泛而谈，真正为党中央决策和国务院编制规划草案提供参考。王晨副委员长出席专题调研动员部署会并作了重要讲话，陈竺、王东明副委员长出席会议并讲话。曹建明、张春贤、沈跃跃、吉炳轩、艾力更·依明巴海、陈竺、王东明、白玛赤林、蔡达峰副委员长分别参加了有关专题调研，并对调研工作给予指导。
　　参加调研的8个专门委员会、1个工作委员会认真制定调研方案，精心组织调研活动，召开有关部门、机构、企业和专家座谈会，分赴部分地方实地考察，在深入调研的基础上认真撰写调研报告，广泛征求各方面意见，反复修改完善。至今年7月中旬，共形成22份专题调研报告，涉及民族、国家安全、财政经济、教科文卫、侨务、环境保护、“三农”、社会保障等诸多领域。
　　总体上看，这次调研有以下特点：一是各单位高度重视，安排部署周密细致。认真学习领会习近平总书记关于各领域工作的新思想、新论断、新要求，把握精髓，指导工作。常委会办公厅和财经委共同牵头制定总体工作方案，有关副委员长及各专门委员会、工作委员会负责同志亲自带队调研，组织精干力量撰写调研报告。二是在疫情防控常态化前提下，做好调研工作。受新冠肺炎疫情的影响，这次调研时间更紧、任务更重，有关专门委员会、工作委员会和常委会办公厅结合疫情防控形势和本单位实际，突出重点、精心组织、加快节奏、加紧工作，同时做到保质保量。三是深入基层，扎实开展调查研究。各单位严格执行中央八项规定精神和疫情防控要求，合理安排出差地点和频次，深入基层、深入群众，全面了解实际情况，掌握大量资料。特别是，积极克服疫情造成的困难，努力创新调研工作方式方法。有的委员会结合常委会执法检查和听取审议专项工作报告一并进行，有的同时委托地方人大和相关专业机构开展调研、提供参阅材料。四是集思广益，广泛征求各方面意见。在调研过程中，各单位主动加强与国务院及其有关部门的沟通联系。有的委员会委托有关行业协会、研究机构和专家就一些重点问题开展专题研究论证，有的研究借鉴发达国家的战略举措与经验启示，有的对近年来相关工作中收集来的意见建议进行梳理，努力使调研报告体现各方面的共识。五是务求实效，形成高质量的调研成果。各单位在调研中坚持问题导向，围绕今后五年我国经济社会发展的重点难点问题深入调查研究，查找问题症结，提出许多具体化、可操作的意见建议。按照专题调研工作方案，22个专题调研报告均以专报形式报常委会领导同志和各有关方面参阅。
　　全国人大常委会开展的这次专题调研，得到了国务院及其有关部门、地方人大和政府、科研单位和专家学者的大力支持和积极配合。22份调研报告凝聚了各方面的意见，是集体智慧的结晶。

　　**二、**专题调研报告的主要内容
　　22份专题调研报告，对相关领域的情况和存在的主要问题进行了梳理，分析研判未来五年面临的形势与挑战，为编制好“十四五”规划纲要提出了大量有价值的意见和建议。
　　民族委员会对“深入推进兴边富民行动情况”进行调研，认为边境地区发展总体仍然滞后，基础设施欠账多，社会事业发展不充分不平衡，人口人才持续流失，边境安全面临新的挑战，需要在“十四五”时期下大力气解决。建议进一步加强党对边境工作的集中统一领导，确保党中央强边固防战略部署落到实处；进一步强化边境地区开发开放顶层设计，把加快边境地区开发开放纳入国家区域发展总体战略；进一步加大对边境地区政策、资金和项目支持力度，更好保障兴边富民行动目标任务落实；进一步提高边境地区各族群众生活水平，推进基本公共服务均等化；进一步推进边疆治理体系和治理能力现代化，加强边境治理法治建设。
　　监察和司法委员会对总体国家安全观和公安执法规范化等两个专题进行调研。关于“落实总体国家安全观问题”的调研认为，各领域国家安全形势依然严峻，重点领域短板凸显，国家安全工作统筹力度有待加强。建议深入贯彻实施[国家安全法](https://www.pkulaw.com/chl/8e9746e69cf66f9cbdfb.html)，形成维护国家安全共识；提升攻坚克难决心，紧抓化解风险战略主动；加大统筹协调力度，凝聚维护国家安全合力；健全国家安全法律体系，推动国家安全法治建设；完善保护保障机制，打造国家安全专业队伍。关于“加强公安执法规范化，推进法治中国建设情况”的调研建议，坚定新时代公安工作的政治方向，进一步提高执法规范化建设的政治站位；统筹中央和地方两个层面，进一步完善法律规范和制度执行体系；坚持规范执法和敢于执法相统一，进一步增强善于执法的能力；夯实标准基础，进一步推动以大数据为核心的信息化建设；推动“软”“硬”并举和“内”“外”共建，进一步强化警务保障水平。
　　财政经济委员会对“产业优化升级和产业链、供应链、价值链布局调整完善”进行调研，认为改革开放特别是党的十八大以来，我国产业结构不断优化升级，产业实力不断增强，产业链、供应链、价值链不断调整完善，但部分环节风险凸显，制造业大而不强且比重下降过快，产业结构和布局仍不合理。“十四五”期间，外部环境深刻变化对我国产业升级和安全形成较大挑战，我国产业优化升级仍有许多有利条件，新一轮科技革命和产业变革带来的机遇与挑战并存。建议把产业链、供应链、价值链布局调整完善上升为国家战略，加强顶层设计，明确目标，统筹规划，合理安排；抓住新一轮科技革命和产业变革契机，推动数字化与经济发展和产业升级深度融合；把发展提升制造业放在更加突出位置，切实减轻企业税费负担，打造市场化、法治化、公平竞争的营商环境；加大科研攻关和知识产权保护力度，以科技创新引领和推动产业优化升级；统筹国内国际两个市场，尽快形成以国内大循环为主体、国内国际双循环相互促进的新发展格局，切实增强抵御外部风险挑战的能力；坚持通过扩大开放促进产业优化升级，在国际竞争中培育新优势。
　　教育科学文化卫生委员会对高等医学教育、创新驱动发展战略、文化产业体系等3个专题进行调研。关于“高等医学教育改革发展”的调研建议，合理确定医学类高等教育招生规模、层次、结构和布局；着力提升医学人才培养质量；加强公共卫生领域人才培养支持力度；进一步深化医教协同改革；加强中医药人才培养；切实增强医学医药研究创新水平。关于“深入实施创新驱动发展战略，建设世界科技强国”的调研建议，充分发挥我国制度优势，完善科研攻关体系；加快推进政府职能深入转变，提升科技资源优化配置和政府创新治理能力；着力提升创新主体能力，强化国家战略科技力量；加强科技创新领域法律制度体系建设，提升法治化水平；推动创新链与产业链深度融合，为经济高质量发展增添动力；拓展科技开放合作，构建全球科技创新命运共同体；进一步加强基础研究，完善组织实施、经费投入、利益分享和人才保障等机制，实现重大原创性成果突破。关于“健全现代文化产业体系和市场体系”的调研建议，加强顶层设计和法治保障，完善以高质量发展为导向的文化经济政策；坚持正确导向，建立健全把社会效益放在首位、社会效益和经济效益相统一的文化创作生产体制机制；坚持创新驱动发展，推动文化产业转型升级；深化文化体制改革，发展壮大市场主体，增强文化产业内生动力；坚持一手抓繁荣、一手抓管理，促进规范文化市场健康有序发展；拓展对外文化贸易广度和深度，加快中华文化“走出去”步伐；总结经验，提升应对重大突发公共事件的能力。
　　华侨委员会对“加强侨务法治建设、充分发挥侨务资源优势助力国家发展战略”开展调研，认为涉侨法律制度和工作机制有待细化，华侨在海外及回国期间的权益保护、侨胞回国创新创业问题不容忽视。建议把握新时代侨务工作新使命，切实加强侨务法治建设；充分发挥侨务资源优势，有力助推国家发展战略实施；始终坚持为侨服务，为侨胞侨企营造良好发展环境；有效开展交流互鉴，积极服务中国特色大国外交；强化涉侨工作合力，努力形成大侨务工作格局。
　　环境与资源保护委员会对黄河流域生态环保、资源综合利用、大气环境质量、水生态环保、海洋生态环保、可再生能源等6个专题进行调研。关于“加强黄河流域生态环境保护”的调研建议，深入学习贯彻落实习近平总书记重要讲话精神，科学编制和实施黄河流域生态保护和高质量发展规划；完善黄河流域生态保护和治理体系；研究制定黄河保护法。关于“资源综合利用”的调研建议，深入学习理解习近平总书记重要讲话精神，牢固树立资源综合高效利用思想；按照全国人大常委会的工作部署，加大力度推动资源综合利用立法研究论证工作；将制定资源综合利用法列入“十四五”规划纲要，明确国家建立资源综合利用的若干具体制度。关于“推动大气环境质量改善”的调研建议，继续攻坚克难，持续打好污染防治攻坚战升级版；坚持依法治污，在法治轨道上推动大气环境质量不断改善；加快结构调整，推动高质量发展；加强制度建设，推进治理体系和能力现代化；强化科技支撑，实现精准科学治污减排。关于“推动水生态环境保护”的调研建议，推动建立“三水”统筹制度体系；加快健全水生态环境保护治理体系；突出抓好城乡饮用水安全保障；因地制宜推进农业农村面源污染防治；强化水生态环境保护法治保障。对“海洋生态环境保护”的调研建议，深入学习贯彻习近平生态文明思想；重点强化陆海统筹，全面提升海洋生态环境保护水平；构建现代海洋产业体系，推进海洋生态恢复和整治修复；充分重视大数据在“十四五”时期的重要支撑和引领作用；加快法律法规标准修改制定，不断完善海洋生态环境保护制度体系；完善协同管理、统筹工作谋划。关于“大力推进可再生能源发展”的调研建议，坚持优先发展可再生能源战略；完善体制机制，统筹解决可再生能源消纳问题；加强统筹协调，综合研究解决补贴资金拖欠问题；健全政策措施，实现可再生能源持续健康发展；坚持科技创新，推动解决发展中出现的问题。
　　农业与农村委员会对粮食安全、高标准农田建设等两个专题进行调研。关于“确保国家粮食安全”的调研建议，稳固粮食综合生产能力的基础；明确粮食结构调整重点任务；深化粮食支持保护政策改革；增强粮食安全科技支撑能力；健全完善粮食安全治理体系。关于“推进高标准农田建设”的调研建议，合理确定建设任务；完善资金筹措机制；强化建后保护管护工作；进一步完善建设管理的体制机制。
　　社会建设委员会对社会保险、养老保险与长护险、医疗保险与生育保险、工伤保险与失业保险等4个专题进行调研。关于“社会保险制度改革和法治建设”的调研建议，调整以劳动关系为标尺的参保准则，探索强制参保，推动社会保险全覆盖；逐步提高基金统筹层次，平衡待遇水平，建立社会保险待遇正常调整机制；健全多渠道筹资机制，完善缴费制度，提升社保基金保值增收能力；围绕风险多发高发重点领域，建立专项制度，提升预防和应对社会风险能力；加强社会保险法治建设，完善法律法规，加大监督和执法力度。关于“养老保险与长护险制度改革与发展”的调研建议，尽快实现养老保险全国统筹，健全筹资和待遇调整机制，适时延迟退休年龄，完善多层次养老体系顶层设计，完善养老保险法律法规；推进和完善长期护理保险制度，加强顶层设计，优化管理体制，建立多渠道筹资机制，规范待遇支付机制，加强长期护理人才建设，完善残疾人长期护理制度。关于“医疗保险与生育保险制度改革与发展”的调研建议，医疗保险要健全重大疾病医保制度，提高医保统筹层次，加快落实异地就医结算制度，优化经办服务管理，推进信息化标准化建设，健全医保基金监管机制，促进多层次医保保障体系发展；生育保险要统一法律规制、实现规范指引，扩大覆盖范围、实现机会公平，强化政府责任、减轻企业负担，统筹区域协调、加强基金管理。关于“工伤保险和失业保险制度改革与发展”的调研建议，工伤保险要坚持法治建设与改革发展并重，预防康复治疗与补偿并重，提高覆盖广度，加快统筹进度，降低保障难度，加大执法力度；失业保险要完善失业保障制度，以结构性改革扩大制度覆盖面，以“放管服”改革提升制度受益面，以省级统筹提升失业保险制度运行效率，以扩大基金支出更好发挥预防失业、促进就业作用。
　　预算工作委员会对税制改革、国有资产管理体制改革等两个专题进行调研。关于“税制改革”专题调研建议，把握好应遵循的基本原则，明确总体目标；在保持宏观税负总体稳定基础上，以合理发挥税制功能作用为导向加强系统规划和改革联动；深化各税种制度改革，提高税制的科学性、规范性；清理规范行政事业性收费和政府性基金等政府非税收入，健全完善政府收入体系；优化征管制度，全面提升征管效率和服务水平；大力加强政府收入法治化建设，落实政府收入法定原则。关于“推进国有资产管理体制改革，有效提升国有资产治理能力”的调研建议，进一步明晰国有资产管理权责；规范对国家出资企业和事业单位的管理方式；建立健全国有资产的国家规划体系；发挥预算在国有资产治理方面的重要作用；加强人大监督、审计监督和社会监督；加强国有资产法律制度建设。

　　**三、**对做好“十四五”规划编制工作的意见和建议
　　编制“十四五”规划，要坚持以习近平新时代中国特色社会主义思想为指导，深入贯彻落实党的十九大和十九届二中、三中、四中、五中全会精神，增强“四个意识”，坚定“四个自信”，做到“两个维护”，统筹推进“五位一体”总体布局，协调推进“四个全面”战略布局，深入贯彻新发展理念，着眼全面建设社会主义现代化国家，紧扣发展不平衡不充分的主要矛盾，统筹谋划中长期战略任务和战略布局，研究确定经济社会发展主要目标指标，有针对性地部署对高质量发展、高效能治理具有牵引性的重大改革和重要政策。结合本次调研，对编制“十四五”规划纲要的总体思路、指导原则提出一些意见和建议：
　　（一）“十四五”规划要立足于我国的国情和实际
　　科学分析当今世界形势，准确把握中国发展大势，是编制好“十四五”规划的前提和基础。“十四五”时期处于“两个一百年”奋斗目标历史交汇期的重要阶段，在实现第一个百年奋斗目标的基础上，将乘势而上开启第二个百年奋斗目标新征程，编制好、实施好“十四五”规划，对于确保未来十五年基本实现社会主义现代化，进而实现第二个百年奋斗目标具有十分重要的意义。当前及未来五年，国际环境持续深刻变化，面临全球化进程受阻、新冠肺炎疫情引发世界经济严重衰退等多重冲击，外部环境总体趋紧，风险挑战前所未有。我国发展已进入新的历史方位，社会主要矛盾发生重大变化，但我国仍处于并将长期处于社会主义初级阶段的基本国情没有变，我国是世界最大发展中国家的国际地位没有变。面对风险挑战，一方面，要一以贯之增强忧患意识，强化底线思维，居安思危，未雨绸缪，宁可把困难想得充分一些，把风险估计得严重一些，做好应对各种局面的思想准备和工作准备。另一方面，要坚持用全面、辩证、长远的眼光看待当前困难、风险和挑战，胸怀两个大局，从世界看中国，从全局看局部，从长期大势认清当前形势。“十四五”规划要尊重经济社会发展规律和我国基本国情，保持国家战略规划的连续性和稳定性，体现中国特色社会主义制度优势，抓住重要战略机遇期，发挥规划的引领和推动作用，变压力为动力，化危机为契机，把我国发展的巨大潜力和强大动能充分释放出来，在危机中育新机、于变局中开新局，承上启下，为实现第二个百年奋斗目标开好局、起好步。
　　（二）坚定不移贯彻落实新发展理念推进高质量发展
　　发展是解决我国一切问题的基础和关键。我国经济已由高速增长阶段转向高质量发展阶段，正处在转变发展方式、优化经济结构、转换增长动力的攻关期，长期积累的结构性、体制性、周期性矛盾和问题没有得到彻底解决，又面临新形势新问题新挑战。“十四五”时期及今后的发展，必须是科学、高质量的发展。要始终保持清醒头脑和战略定力，着力转方式调结构，深入贯彻崇尚创新、注重协调、倡导绿色、厚植开放、推进共享的新发展理念，按照高质量发展和建设现代化经济体系的要求，坚持以供给侧结构性改革为主线，统筹推进稳增长、促改革、调结构、惠民生、防风险、保稳定各项工作，努力实现更高质量、更有效率、更加公平、更可持续的发展。“十四五”期间要着力解决长期制约经济社会平稳健康发展的深层次矛盾和问题，力争取得突破性进展。22个调研题目都体现了贯彻新发展理念这一原则，提出了很多具体的意见和建议。
　　（三）进一步落实以人民为中心的发展思想
　　坚持以人民为中心的发展思想，坚持人民至上，紧紧依靠人民、不断造福人民、牢牢植根人民，做到发展为了人民、发展依靠人民、发展成果由人民共享，这充分体现了中国共产党的理想信念、性质宗旨和初心使命，也是对党的奋斗历程和实践经验的深刻总结，必须贯彻落实到各项决策部署和实际工作之中。在经济面临较大下行压力的情况下，更要坚持以人民为中心，持续改善人民福祉。编制“十四五”规划要从人民群众最关心、最直接、最现实的根本利益出发，多谋民生之利、多解民生之忧。围绕民生大事难事急事，聚焦重点人群，关注重点问题，坚持尽力而为、量力而行，加快补齐就业、教育、社保、医疗、住房、养老、环境污染、食品安全、社会治安等方面的明显短板，努力实现幼有所育、学有所教、劳有所得、病有所医、老有所养、住有所居、弱有所扶，切实提高人民群众的获得感、幸福感和安全感。特别是在新冠肺炎疫情冲击下，要加大基本民生兜底保障力度，着力做好困难群体基本生活保障。进一步保障重点群体就业，防止出现大规模失业，对生活困难的失业人员及家庭按规定纳入最低生活保障、临时救助等社会救助范围。尽快补齐公共卫生短板，加强公共卫生体系和应急物资保障体系建设。
　　（四）继续推进改革开放增强发展活力与后劲
　　形势越是困难，越要坚持深化改革。开启全面建设社会主义现代化国家新征程，必须发挥好改革的突破和先导作用，向改革要动力、要活力、要效益、要发展后劲，依靠改革应对变局、开拓新局。要把党的十八届三中全会以来全面深化改革任务同完成“十三五”规划主要目标、决战决胜脱贫攻坚和全面建成小康社会结合起来，有针对性地推进关键领域改革。同时，认真谋划“十四五”时期改革工作，更加注重制度和治理体系建设，更多解决深层次体制机制问题，扎实推进国家治理体系和治理能力现代化。实施更大范围、更宽领域、更深层次的全面开放，围绕高质量推进“一带一路”建设，加快制度型开放，构筑全面开放新格局，培育新形势下国际合作与竞争新优势，积极主动参与国际宏观政策协调和国际经贸规则制定，提升我国在国际事务中的话语权，形成以开放促改革促发展的新局面。
　　（五）全面贯彻落实总体国家安全观维护国家安全
　　总体国家安全观是新时代坚持和发展中国特色社会主义的十四条基本方略之一，也是党和人民维护国家安全的根本遵循。“十四五”时期要全面贯彻落实总体国家安全观，牢固树立安全发展理念，全面提高抵御各种风险的能力。在事关国家安全重要战略和重要工作上，要坚持继承和发展创新的辩证统一，处理好变与不变的关系，加强分析研判和预警监测，牢牢守住不发生系统性风险的底线。聚焦粮食、能源、金融、网络、生物、公共卫生等重点领域风险，严厉打击恐怖主义、分裂主义和极端主义犯罪活动，加大重点领域国家安全保障能力投入，制定不同情境下的应对预案，确保社会大局稳定，全方位提升人民群众安全感。
　　（六）扎实推进国家治理体系和治理能力现代化
　　“十四五”时期要继续坚持和完善中国特色社会主义制度和社会主义市场经济体制，推进国家治理体系和治理能力现代化。要充分认识工作中的不足和薄弱环节，深入研究和解决存在的突出问题，把制度优势转化为管理经济社会事务的效能。一些专门委员会和工作委员会在调研报告中指出，要加快推动相关立法进程，以适应改革和发展的需要。比如，边疆治理方面，制定修改陆地边界法、[国境卫生检疫法](https://www.pkulaw.com/chl/fb552971923850bbbdfb.html)等法律；国家安全和经济安全方面，抓紧出台数据安全法、个人信息保护法、生物安全法、出口管制法以及维护产业链供应链稳定安全和产业救济方面的法律法规等；公安执法方面，修改[人民警察法](https://www.pkulaw.com/chl/24c23cf279e4fc8abdfb.html)、[治安管理处罚法](https://www.pkulaw.com/chl/1095cd22312af2f3bdfb.html)、[道路交通安全法](https://www.pkulaw.com/chl/6da39f72c1df6082bdfb.html)，制定看守所法等；财政经济方面，加强税收立法，完善国有资产立法，修订[反垄断法](https://www.pkulaw.com/chl/503218397088141cbdfb.html)，积极推动[外商投资法](https://www.pkulaw.com/chl/6a88714068b3724dbdfb.html)配套法规尽快出台；科技文化方面，加快修订科技进步法、[专利法](https://www.pkulaw.com/chl/657ae1bc83599617bdfb.html)、[著作权法](https://www.pkulaw.com/chl/b3e0f208f7fa69eabdfb.html)，制定文化产业促进法等；环境资源方面，研究制定黄河保护法，修订[海洋环境保护法](https://www.pkulaw.com/chl/174b2b5043ca235dbdfb.html)，加快推动大气环境保护配套法规的制修订；农业农村方面，制定乡村振兴促进法；社会保障方面，制定和修改[社会保险法](https://www.pkulaw.com/chl/4449c7967540e761bdfb.html)、医疗保障法、基本养老保险条例、基本医疗保险条例和[失业保险条例](https://www.pkulaw.com/chl/83a0e199717d4f59bdfb.html)等。
　　以上情况，特此报告。

© 北大法宝：（[www.pkulaw.com](https://www.pkulaw.com/)) 专业提供法律信息、法学知识和法律软件领域各类解决方案。北大法宝为您提供丰富的参考资料，正式引用法规条文时请与标准文本核对。
欢迎查看所有[产品和服务](https://www.pkulaw.com/helps/2.html)。法宝快讯：[如何快速找到您需要的检索结果？](https://www.pkulaw.com/helps/70.html)[法宝](https://www.pkulaw.com/helps/69.html)V6有何新特色？

扫描二维码阅读原文

**原文链接：**<https://www.pkulaw.com/chl/5f91ac945b1c07ccbdfb.html>
